# Anti‐cancer potentials of *Gynura procumbens* leaves extract against two canine mammary cancer cell lines

**DOI:** 10.1002/vms3.684

**Published:** 2021-12-09

**Authors:** Usuma Jermnak, Wachiraphan Supsavhad, Sunee Kunakornsawat, Tassanee Jaroensong, Piyajit Watcharasit, Daranee Visitnonthachai, Selapoom Pairor, Napasorn Phaochoosak

**Affiliations:** ^1^ Department of Pharmacology Faculty of Veterinary Medicine Kasetsart University Bangkok Thailand; ^2^ Department of Pathology Faculty of Veterinary Medicine Kasetsart University Bangkok Thailand; ^3^ Department of Companion Animal Clinical Sciences Faculty of Veterinary Medicine Kasetsart University Bangkok Thailand; ^4^ Laboratory of Pharmacology Chulabhorn Research Institute Bangkok Thailand

**Keywords:** anti‐cancer properties, canine mammary cancer, EGFR signalling pathway, *Gynura procumbens*, leaves extract

## Abstract

**Background:**

The anti‐cancer effects of *Gynura procumbens* leaves extract (GPE) have been reported in various human cancers. However, the anti‐cancer effects and molecular mechanisms of this extract on canine mammary cancer (CMC) have not yet been elucidated.

**Objectives:**

The main goal of this study was to investigate the anti‐cancer properties of GPE against two CMC cell lines (CHMp‐13a and CHMp‐5b).

**Methods:**

The GP leaves were extracted with 80% ethanol. Anti‐cancer potentials of GPE on CHMp‐13a and CHMp‐5b cancer cell lines using dimethyl‐2‐thiazolyl‐2,5‐diphenyl‐2H‐tetrazolium bromide (MTT), wound healing, transwell migration, and caspase 3/7 activity assays were evaluated. The mRNA expression levels of two oncogenes: epidermal growth factor receptor (*EGFR*) and twist family bHLH transcription factor 1 (*TWIST*) and one tumour suppressor gene: phosphatase and tensin homolog (*PTEN*) in these cell lines were determined by quantitative real‐time PCR (qRT‐PCR). In addition, The EGFR and PTEN protein levels as well as protein kinase B (AKT) and extracellular signal‐regulated kinase 1/2 (ERK1/2) phosphorylation levels expression were also evaluated by western blot analysis.

**Results:**

The results showed that GPE caused a significant concentration‐ and time‐dependent reduction in cell proliferation of both CHMp‐13a and CHMp‐5b cells, detected by MTT assays. This extract also significantly suppressed cancer cell migration in both cell lines, tested by wound healing and transwell migration assays. Additionally, the increase in caspase 3/7 activity observed in both CMC cell treated with GPE suggests that GPE induced caspase 3/7 dependent apoptosis. Moreover, GPE significantly decreased EGFR mRNA and protein expression levels compared to control in both cell lines in a dose‐dependent manner.

**Conclusion:**

These findings emphasized that GPE has an in vitro anti‐cancer activity against CMC by inhibiting EGFR signalling pathway. Thus, GPE may serve as an alternative therapy in CMC with high EGFR expression.

## Graphical abstract

The anti‐cancer properties of *Gynura procumbens* leaves extract were investigated in two canine mammary cancer cell lines. The results indicate that this herbal extract inhibits the cell proliferation and migration, and induces apoptosis of both cancer cell lines via the inhibition of EGFR signalling pathway. Therefore, *Gynura procumbens* leaves extract may serve as an alternative therapy in mammary cancer in dogs with high EGFR expression.

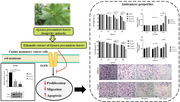

## 1 INTRODUCTION

Mammary tumours are the most common tumours in female dogs. Approximately 50% of these tumours are malignant (Salas et al., 2015). These tumours can originate from various types of mammary tissues (Kanae et al., 2006). Several risk factors for tumour development have been identified, including hormonal, genetic, and nutritional factors (Beauvais et al., 2012; Benavente et al., 2016; Sorenmo et al., 2009). Similarities between human and dog mammary tumours, including hormonal dependence, spontaneous development, metastatic behaviour, and the pattern of neoangiogenesis, have been reported (Bernstein, 2002; Di Cerbo et al., 2014; Sultan & Ganaie, 2018). Moreover, the pathogenesis and progression of this cancer are related to a number of critical genes and proteins that control cell proliferation, migration, invasion, and apoptosis (Santos et al., 2013). In canine mammary cancer (CMC), changes in the expression of many genes and protein levels, such as epidermal growth factor receptor (EGFR), phosphatase and tensin homolog (PTEN), and TWIST, have been reported (Beirão et al., 2015; Qiu et al., 2008; Silva et al., 2014). The positive correlation between tumour progression and EGFR expression in the CMCs has previously been demonstrated (Masuda et al., 2012). The up‐regulation of PTEN expression can induce CMC cell apoptosis (Cantley & Neel, 1999), while the up‐regulation of TWIST expression can enhance the metastatic ability of mammary cancer (Beirão et al., 2015). The activation of EGFR results in the activation of downstream signalling pathways including the phosphatidylinositol 3‐kinase (PI3K)/AKT pathway and extracellular signal‐regulated kinase 1/2 (ERK1/2) pathway. These pathways play a key role in regulating the proliferation and controlling the survival and metastatic potential of tumour cells (Dent, 2014; Jarvis et al., 1997; Toulany et al., 2014). Thus, EGFR and its downstream signalling molecules have appeared as promising targets for cancer therapy in human and dogs.

At present, the most reliable treatment of CMC is surgical removal, consisting of removing the tumour and its regional lymph nodes (Cassali et al., 2011; Kaszak et al., 2018). Even though various chemotherapeutic agents have been applied in CMC treatment, they have significant disadvantages, including the price, highly adverse effects, and limited information on the efficacy (Sleeckx et al., 2011; Tran et al., 2016). Plant extracts have been considered as an effective approach to control various diseases including cancers. The effectiveness of plant extracts and their active components on various human cancer cell lines has been reported (González‐Vallinas et al., 2013). However, only a few studies have been conducted in canine cancer cell lines (Gultiken et al., 2015; Levine et al., 2017; Wakshlag & Balkman, 2010).

*Gynura procumbens* (GP) is a medicinal plant commonly found in tropical Asian countries, especially in Indonesia, Malaysia, and Thailand. This plant has been used as a traditional medicine for cancer, inflammation, diabetes mellitus, bacterial, and viral infection treatments (Ashraf, 2019; Iskander et al., 2002; Tan et al., 2016). The leaves of GP are safely consumed without toxic effects (Rosidah et al., 2009; Teoh et al., 2016). Over the last few years, intensive studies related to GP have provided extensive scientific evidence of its therapeutic potential, particularly anti‐cancer properties, on human, as well as rodent cancer cell lines (Afandi et al., 2014; Ashraf et al., 2020; Gofur et al., 2015;). The proliferation inhibiting effect of *Gynura procumbens* leaves extract (GPE) on human breast cancer and rodent hepatic cancer has been reported (Hew et al., 2013; Nisa et al., 2012). However, the anti‐cancer effect of this plant extract on CMC is unclear. In addition, the molecular pathway that may contribute to its anti‐carcinogenic effects has not yet been elucidated.

In this study, the anti‐proliferation, anti‐migration, and apoptotic effects of GPE were investigated in two CMC cell lines, CHMp‐13a and CHMp‐5b. The mRNA expression levels of *PTEN*, *EGFR*, and *TWIST* were relatively quantified on these two CMC cell lines. In addition, the effects of GPE on the protein (PTEN and EGFR) and phosphorylation (AKT and ERK1/2) level expression were also evaluated in these cell lines.

## 2 MATERIALS AND METHODS

### 2.1 Plant material and extraction

Leaves of GP were collected from the local area of Nonthaburi province, Thailand. A voucher number of the specimen (TTM‐c No.1000629) was deposited at the Herbarium of Thai Traditional Medicine Research Institute, Bangkok, Thailand. Two kilograms of fresh leaves were cleaned by rinsing with distilled water and dried in an oven at 60°C, then ground into powdered form. One gram of powdered leaves of GP was extracted with 20 ml of 80% ethanol at room temperature for 3 h. Then, the extract was filtered through Whatman No. 1 filter paper and evaporated to dryness under reduced pressure on an R300 Buchi rotary evaporator (Buchi, Flawil, Switzerland) at 40°C. Then the extract was dissolved in 100 mg/ml dimethyl sulfoxide (DMSO). The final concentration of DMSO in culture medium was diluted to 0.1% (v/v).

### 2.2 Cell lines and cell culture

The CMC cell lines, CHMp‐13a (a low‐grade invasive carcinoma) and CHMp‐5b (a high‐grade invasive carcinoma) were maintained in RPMI‐1640 containing l‐glutamine medium (Corning, Corning, NY, USA) and supplemented with 10% fetal bovine serum (FBS) (Invitrogen, Carlsbad, CA, USA), 5 mg/L penicillin‐streptomycin (Invitrogen) at 37°C in 5% CO_2_ atmosphere. The medium was refreshed every 2–3 days. After 80% confluence, cells were harvested using 0.25% trypsin‐EDTA (Corning) and sub‐cultured.

The mycoplasma status in both CHMp‐13a and CHMp‐5b cell lines were tested using PCR‐based detection methods. For detection of mycoplasma, 500 μl of CHMp‐13a and CHMp‐5b cell culture medium were taken after a culture period of 2 days without changing the medium. The mycoplasma status was investigated using PCR‐based method as described by Criado‐Fornelio et al. (2003). The total DNA from cell culture samples were extracted using Genomic DNA Mini Kit for blood/cultured cells (Geneaid, Biotech, Taiwan) according to the manufacturer's protocol. PCR reaction mixtures were prepared in a total volume of 25 μl containing, 1× PCR buffer, 50 μM each of dNTP, 1 μl of each primer, 0.5 μl Taq DNA polymerase, 1.5 mM MgCl_2_, and 1 μl of mycoplasma genomic DNA as a positive control. Thermal profiles are as follows: initial denaturation at 95°C for 5 min, 40 cycles consisting of denaturation at 95°C for 30 s, annealing at 60°C for 30 s, and extension at 72°C for 30 s, followed by a final extension at 72°C for 10 min. Both cell lines used in this study were free from mycoplasma contamination.

### 2.3 Cell proliferation test

The cytotoxicity effect of GPE was studied by MTT assay. The CHMp‐13a and CHMp‐5b cells (7 × 10^3^ cells/well) were seeded into 96‐well plates and allowed to grow for 24 h. They were then treated with GPE at various concentrations (40, 80, and 160 μg/ml) for 0, 17, 21, and 28 h, whereas the negative control group was treated with the culture medium containing DMSO (0.1%). After incubation, 10 μl of MTT solution was added to each well and the plate was further incubated for 4 h. Then, 100 μl of solubilization solution was added to each well to solubilize water insoluble purple formazan crystals, followed by overnight incubation at 37°C in 5% CO_2_. The absorbance at 570 nm was measured using a Synergy H1 hybrid multi‐mode microplate reader (BioTek, Winooski, VT, USA). The experiment was performed in three replicates. The results of absorbance values of treated cells were expressed as percentage of cell viability relatively to untreated control cells (considered as 100% viability).

### 2.4 Cell migration assay

The effect of GPE on cell migration was examined by a two‐dimensional wound healing assay. The CHMp‐13a and CHMp‐5b cells were seeded in a 6‐well plate at 2 × 10^4^ cells/well and allowed to grow for 48 h (confluency 100%). Cells were scratched using a sterile 1 ml pipette tip. They were subsequently treated with different concentrations of GPE (40, 80, and 160 μg/ml) or the culture medium containing DMSO (0.1%) for 21 h. Images of the scratch were taken at 0, 6, 12, and 21 h after treatment using phase contrast microscopy. The wound areas were measured by using NIS‐Elements D imaging software program (Nikon, Tokyo, Japan). The wound area percentage was calculated according to the following formula:

(1) $$\text{Wound}\;\text{area}\;\text{percent}\left(\%\right)=\frac{\text{Area}\;\text{between}\;\text{cells}\;\;\left(6,\;12,\;\mathrm{or}\;21\;h\right)}{\text{Area}\;\text{between}\;\text{cells}\;\;\left(0\;h\right)}\times \;100.\;$$

### 2.5 Transwell migration assay

Cell migration was also quantified by transwell assays. Transwell filters (8 μm pore size; Costar, Corning, NY, USA) were placed in 24‐well plates. The CHMp‐13a and CHMp‐5b cells were seeded onto the filters at a density of 2.5 × 10^3^ cells/well with 5% FBS‐RPMI 1640 containing various concentrations of GPE (0, 40, 80, and 160 μg/ml). The medium containing 10% FBS was then added to the lower part of the chamber, and the cells were incubated for 21 h. After 21 h incubation, cells remaining on the upper surface of membrane were removed by gently scrubbing with cotton swab moistened with the medium. The cells that had migrated to the underside of the membrane were fixed with 100% methanol for 1 min and stained with Wright‐Giemsa for 15 min, respectively. The number of migrated cells per 40× magnification was determined by averaging the cell counts in five random fields of each transwell membrane.

### 2.6 Caspase 3/7 activity assay

The CHMp‐13a and CHMp‐5b cells were cultured in white opaque tissue culture plates (Costar) at a density of 3 × 10^3^ cells/well and allowed to grow for 24 h. They were then treated with GPE at various concentrations (0, 80, and 160 μg/ml) for 0, 6, 12, and 28 h. respectively. After incubation, 100 μl of caspase‐glo 3/7 reagents (Promega Corp., Madison, WI, USA) was added to the designated wells. The contents of wells were gently mixed at 300 rpm (Rotamax 120; Heidolph, GmbH & CO. KG, Germany) for 1 min and incubated at room temperature for 1 h. The luminescence was determined using a Synergy H1 hybrid multi‐mode microplate reader. Caspase 3/7 activity was expressed as percentage of control. The experiment was performed in four replicates.

### 2.7 RNA isolation and quantitative real‐time PCR

After treatment of the cells with the GPE at concentrations of 0, 40, 80, and 160 μg/ml for 28 h, the total RNA was isolated using GeneJet RNA Purification kit (Thermo Fisher Scientific, Waltham, MA, USA) according to the manufacturer's protocol for adherent tissue culture cells. Total RNA (0.5 μg) was reverse transcribed using the Superscript III First Strand Synthesis system for reverse transcription PCR (Invitrogen) at 65°C for 5 min, 50°C for 50 min, 85°C for 5 min, and 37°C for 5 min. The relative mRNA expression levels of canine *PTEN, EGFR*, and *TWIST* were normalized to canine glyceraldehyde 3‐phosphate dehydrogenase (*GAPDH*) using G‐Storm GS482 PCR thermal cycler (Gene Technologies, Somerset, UK) (Elshafae et al., 2016). Primer sequences for canine *GAPDH, PTEN, EGFR*, and *TWIST* are listed in Table 1. The relative mRNA expression levels were calculated using the 2^–∆∆^ *^Cq^* method. The transcriptional level of *PTEN, EGFR*, and *TWIST* genes were determined in six replicates by quantitative real‐time PCR (qRT‐PCR) using a CFX 96 Touch Real time PCR Detection System (Bio‐Rad, Hercules, CA, USA). The qRT‐PCR was performed using the reaction mixture containing 2 μl of cDNA template, 10 μl of QuantiTect SYBR Green PCR kit (Qiagen, Hilden, Germany), and 1 μl of each primer. The qRT‐PCR protocol was as follows: initial activation at 95°C for 15 min; denaturation at 94°C for 15 s, annealing at 60°C for 30 s, extension at 72°C for 30 s, and fluorescent data acquisitions at 72°C for 1 min (40 cycles).

**TABLE 1:** Primer sequences for quantitative real‐time PCR (qRT‐PCR)

Gene	Primer
*GAPDH*	Forward primer 5′‐CCCACTCTTCCACCTTCGAC‐3′ Reverse primer 5′‐AGCCAAATTCATTGTCATACCAGG‐3′
*PTEN*	Forward primer 5′‐AAAGCTGGAAAGGGACGAACTGGTG‐3′ Reverse primer 5′‐ACACATAGCGCCTCTGACTGGGAAT‐3′
*EGFR*	Forward primer 5′‐ CGAGCACAAGGACAACATCG −3′ Reverse primer 5′‐ CTCCACACATCGCTTTGGTG −3′
*TWIST*	Forward primer 5′‐GGCAGGGCCGGAGACCTAGATG‐3′ Reverse primer 5′‐TCCACGGGCCTGTCTCGCTT‐3′

### 2.8 Western blot analysis

Following treatment of the CHMp‐13a and CHMp‐5b cells with the GPE at concentrations of 0, 80, and 160 μg/ml for 28 h, cells were harvested, washed with ice‐cold PBS cells, and lysed with ice‐cold RIPA buffer (20 mM Tris‐HCl pH 7.5, 150 mM NaCl, 1 mM Na_2_EDTA, 1 mM EGTA, 1% NP‐40, 1% sodium deoxycholate, 2.5 mM sodium pyrophosphate, 1 mM b‐glycerophosphate, 1 mM Na_3_VO_4_, and 1 μg/ml leupeptin) in the presence of a protease inhibitor mixture and phosphatase inhibitor cocktail (Cell Signalling Technology, Danvers, MA, USA). The cell lysates were sonicated then centrifuged at 14,000× g for 10 min at 4°C to remove insoluble material, and the resulting supernatants were stored at −80°C until use. The protein concentration was determined with Pierce BCA protein assay kit (Thermo Fisher Scientific). For western blotting, the cell lysate was mixed with 2× Laemmli sample buffer (125 mM Tris pH 6.8, 20% glycerol, 4% SDS, 0.004% bromophenol blue, and 5% β‐mercaptoethanol), and boiled for 5 min. Samples (40 μg protein each) were loaded in SDS‐PAGE and transferred to nitrocellulose membranes. The membranes were probed with primary antibody overnight at 4°C with agitation, followed by incubation with an appropriate secondary antibody conjugated with horseradish peroxidase (Bio‐Rad). Protein bands were visualized using Clarity Western Enhanced Chemiluminescence (Bio‐Rad), followed by exposure to X‐ray Film (Fuji Film, Tokyo, Japan). Antibodies used were rabbit anti‐phospho‐ERK1/2 (#9101; 1:2000), rabbit anti‐ERK1/2 (#9107; 1:4000), rabbit anti‐phospho‐AKT (S473) (#9271; 1:1000), rabbit anti‐AKT (#2920; 1:4000), rabbit anti‐EGFR (#4267; 1:1000), rabbit anti‐PTEN (#9188; 1:1000) (Cell Signalling Technology), and anti‐β‐actin (#A5316; 1:30,000) (Sigma‐Aldrich, St. Louis, MO, USA). Protein band density was analyzed using densitometer with Image Lab software (Bio‐Rad). The expression levels of EGFR and PTEN were quantitated and normalized with β‐actin derived from the same blot, and expressed as percentage of control. The phosphorylation levels of AKT and ERK1/2 were quantitated and normalized to the total AKT and ERK1/2, respectively. All antibodies react with canine proteins. Anti‐phospho‐AKT (S473) (CST#9271), anti‐PTEN (CST#9188) and anti‐β‐actin (Sigma‐Aldrich #A5316) antibodies were tested by the manufacturer. Anti‐AKT (CST#2920), anti‐phospho‐ERK1/2 (CST#9101), anti‐ERK1/2 (CST#9107), and anti‐EGFR (CST#4267) have been reported in Kake et al. (2017) and Jin et al. (2016), respectively.

### 2.9 Statistical analysis

The data generated in this study are expressed as mean ± SD of at least three independent experiments. Statistical differences between two groups were analyzed using Kruskal Wallis followed by Dunn's multiple comparisons post hoc tests. Statistical analysis was performed using GraphPad Prism 6.0 (GraphPad Software, Inc., La Jolla, CA, USA). *p* < 0.05 was considered to indicate a statistically significant difference.

## 3 RESULTS

### 3.1 Anti‐proliferation effect of GPE on CHMp‐13a and CHMp‐5b cells

**FIGURE 1:**
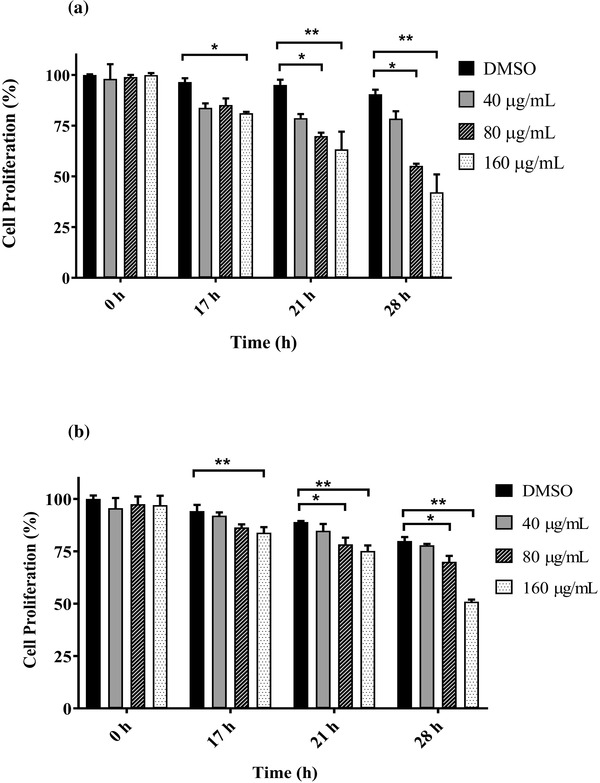
Effects of *Gynura procumbens* leaves extract (GPE) on CHMp‐13a (a) and CHMp‐5b (b) cell proliferation using the MTT assay. Cells (7 × 10^3^ cells/well) were incubated for 17, 21, and 28 h with four different concentrations (0, 40, 80, and 160 μg/ml) of GPE. Each data point indicates the mean of three separate experiments ± SD. Statistical differences were analyzed using Kruskal Wallis followed by Dunn's multiple comparisons post hoc tests. Significant differences from the control are indicated: ^∗^ *p* < 0.05; ^∗∗^ *p* < 0.01

The MTT assay was used to examine the growth inhibition effect of GPE in CHMp‐13a and CHMp‐5b cells. The total cell numbers of CHMp‐13a and CHMp‐5b cells treated with 80 and 160 μg/ml of GPE were significantly reduced by 10%–25% compared with the control group after 21 h of treatment. The GPE at a concentration of 80 μg/ml reduced the cell viability of CHMp‐13a and CHMp‐5b cells by 35% and 10%, whereas at a concentration of 160 μg/ml, the cell viability reduced to almost 50% and 30% with comparing the control treatment after 28 h of exposure, respectively (Figure 1a,b). This result clearly demonstrates that GPE caused a significant concentration and time dependent reduction in cell proliferation of both CHMp‐13a and CHMp‐5b cells.

### 3.2 Anti‐migration effect of GPE on CHMp‐13a and CHMp‐5b cells

**FIGURE 2:**
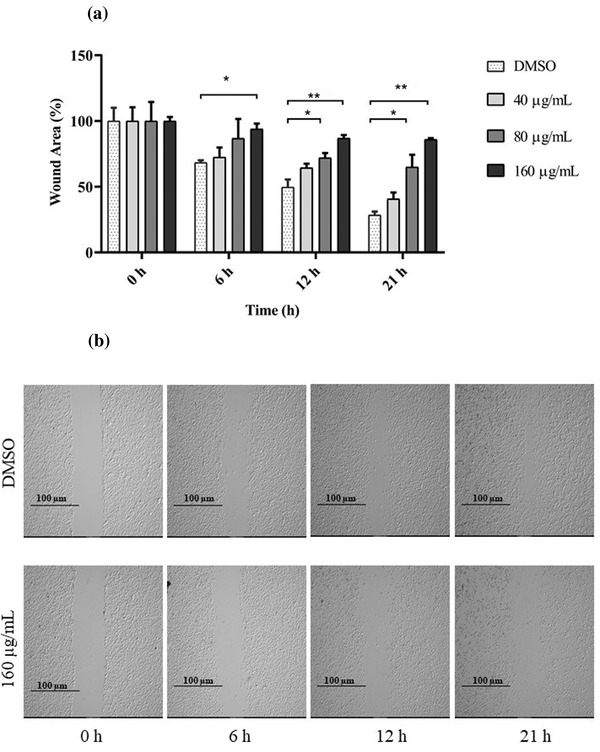
Effects of *Gynura procumbens* leaves extract (GPE) on CHMp‐13a cell migration was measured by the wound healing assay. CHMp‐13a cells were treated with four different concentrations (0, 40, 80, and 160 μg/ml) of GPE at 0, 6, 12, and 21 h. (a) The percentage wound area was measured by comparing the change in wound area to that of the untreated group at 0 h. Each data point indicates the mean of three separate experiments ± SD. Statistical differences were analyzed using Kruskal Wallis followed by Dunn's multiple comparisons post hoc tests. Significant differences from the control are indicated: ^∗^ *p* < 0.05; ^∗∗^ *p* < 0.01. (b) Cell migration was monitored by phase contrast microscopy

**FIGURE 3:**
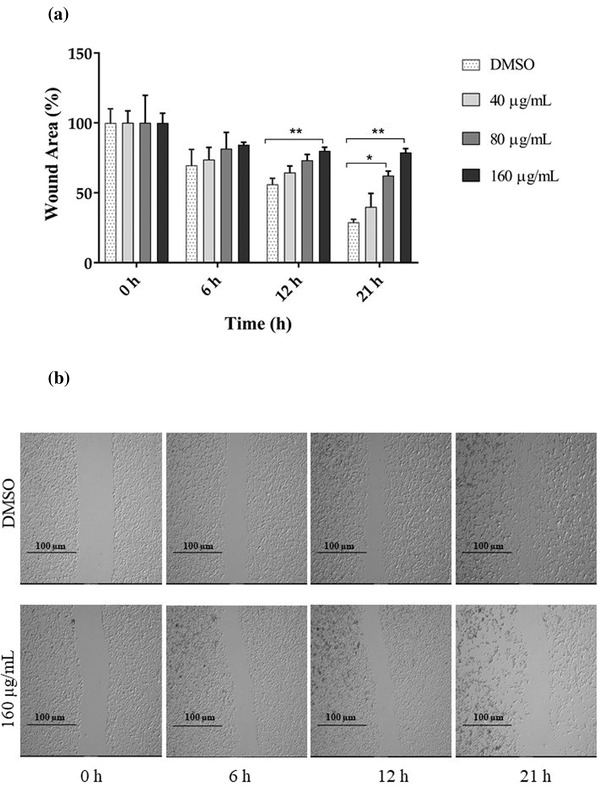
Effects of *Gynura procumbens* leaves extract (GPE) on CHMp‐5b cell migration was measured by the wound healing assay. CHMp‐5b cells were treated with four different concentrations (0, 40, 80, and 160 μg/ml) of GPE at 0, 6, 12, and 21 h. (a) The percentage wound area was measured by comparing the change in wound area to that of the untreated group at 0 h. Each data point indicates the mean of three separate experiments ± SD. Statistical differences were analyzed using Kruskal Wallis followed by Dunn's multiple comparisons post hoc tests. Significant differences from the control are indicated: ^∗^ *p* < 0.05; ^∗∗^ *p* < 0.01. (b) Cell migration was monitored by phase contrast microscopy

**FIGURE 4:**
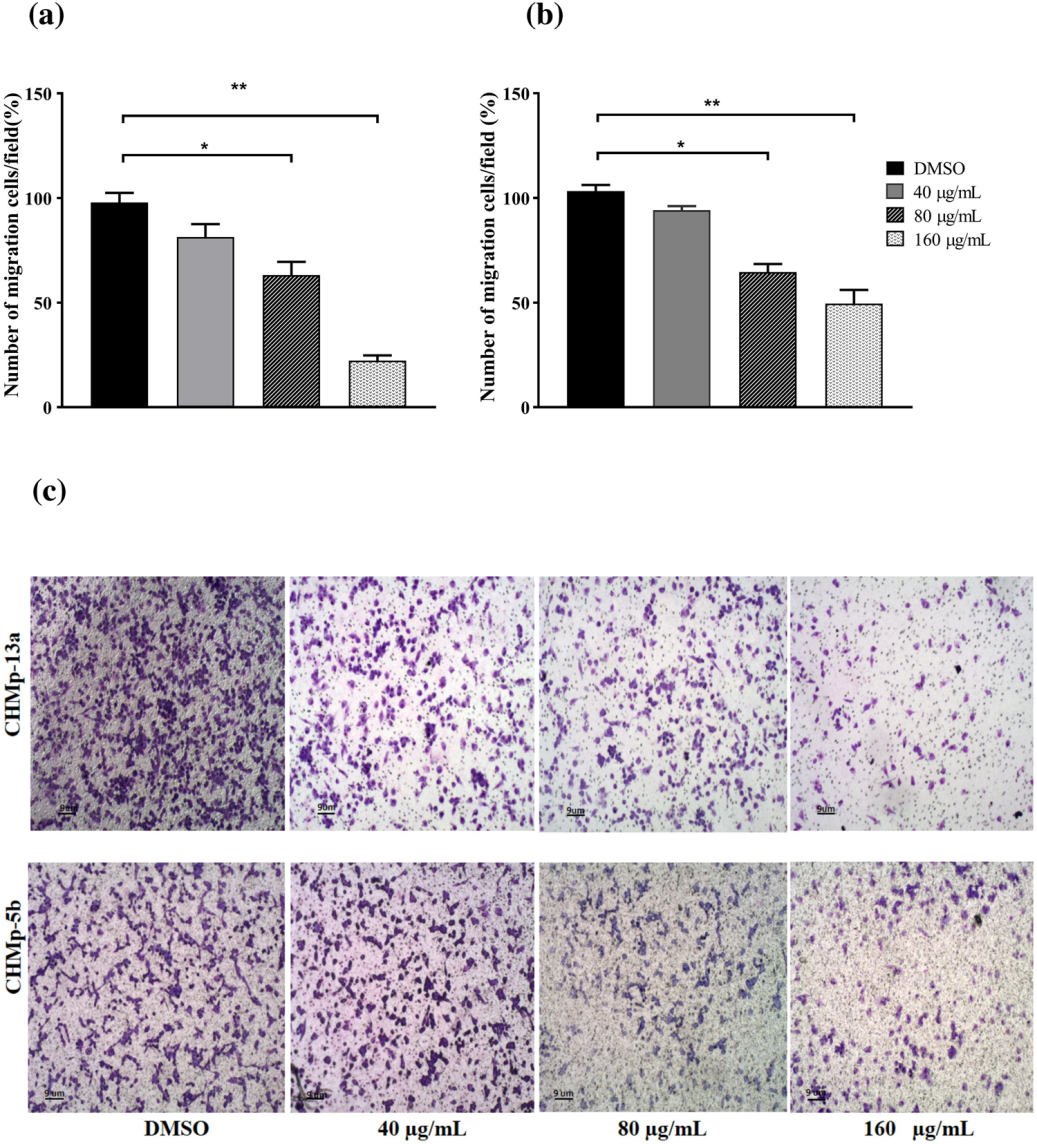
Effects of *Gynura procumbens* leaves extract (GPE) on CHMp‐13a and CHMp‐5b cells migration was measured by the transwell migration assay. Cells were treated with four different concentrations (0, 40, 80, and 160 μg/ml) of GPE at 21 h. (a and b) The percentage of number of CHMp‐13a and CHMp‐5b cells migration was measured by comparing the number of migrating cells after treatment with different concentrations of GPE to that of the control group. Each data point indicates the mean of three separate experiments ± SD. Statistical differences were analyzed using Kruskal Wallis followed by Dunn's multiple comparisons post hoc tests. Significant differences from the control are indicated: ^∗^ *p* < 0.05; ^∗∗^ *p* < 0.01. (c) Images represent CHMp‐13a and CHMp‐5b cells migration to the underside of transwell membranes following treatment with 0, 40, 80, and 160 μg/ml of GPE at 21 h (original magnification, 4×)

To determine whether GPE inhibits CHMp‐13a and CHMp‐5b cell migration, the wound healing and transwell migration assays were performed. In the wound healing assay, we found that the percentage of wound areas in CHMp‐13a and CHMp‐5b cells supplemented with 80 and 160 μg/ml of GPE for 12 and 21 h were significantly greater than the untreated cells (Figures 2 and 3). Likewise, GPE significantly inhibited both CMC cell migration in a 21 h transwell migration assay in comparison with untreated control cells (Figure 4). Treatment with 80 and 160 μg/ml of GPE inhibited CHMp‐13a cells migration by 36% and 77%, respectively, whereas the same concentrations of GPE inhibited CHMp‐5b cells migration by 35% and 50%, respectively. The wound healing and transwell migration assays both indicated that GPE significantly suppresses the migration of both CMC cells in a dose‐dependent manner.

### 3.3 Effect of GPE on apoptosis of CHMp‐13a and CHMp‐5b cells

**FIGURE 5:**
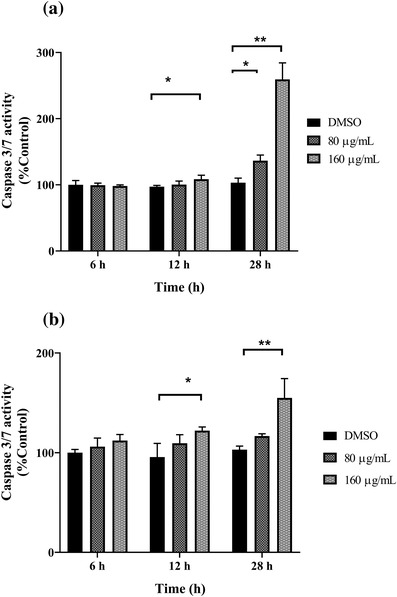
Effects of *Gynura procumbens* leaves extract (GPE) on the caspase 3/7 activation in CHMp‐13a (a) and CHMp‐5b (b) cells were estimated using luminescence analysis. Cells were treated with three different concentrations (0, 80, and 160 μg/ml) of GPE at 0, 6, 12, and 28 h. Caspase 3/7 activity was expressed as percentage of control. Each data point indicates the mean of four separate experiments ± SD. Statistical differences were analyzed using Kruskal Wallis followed by Dunn's multiple comparisons post hoc tests. Significant differences from the control are indicated: ^∗^ *p* < 0.05; ^∗∗^ *p* < 0.01

The caspase 3/7 activity assay was performed to determine if GPE could induce caspase 3/7‐dependent apoptosis in the CHMp‐13a and CHMp‐5b cells at 28 h. The results showed that the extract induced concentration‐dependent increases in caspase 3/7 activity in CHMp‐13a and CHMp‐5b cells. The GPE at a concentration of 160 μg/ml increased the caspase 3/7 activation of CHMp‐13a and CHMp‐5b cells by 2.7 and 1.5 folds when compared to the control group after 28 h of treatment, respectively (Figure 5).

### 3.4 Effect of GPE on PTEN, EGFR, and TWIST relative mRNA expression levels in CHMp‐13a and CHMp‐5b cells

CHMp‐13a and CHMp‐5b cells were treated with culture medium supplemented with different concentrations (0, 40, 80, and 160 μg/ml) of GPE for 28 h. The mRNA expression levels of canine *PTEN*, *EGFR*, and *TWIST* genes were measured using the qRT‐PCR. Relative abundance of mRNA was obtained by normalization to the expression level of canine GAPDH mRNA.

**FIGURE 6:**
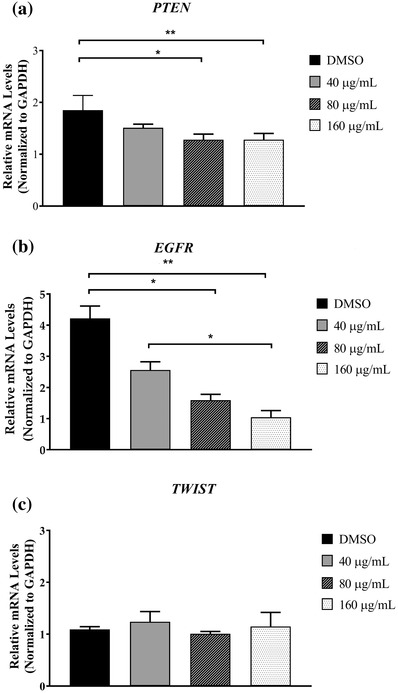
Effects of *Gynura procumbens* leaves extract (GPE) on *PTEN, EGFR*, and *TWIST* gene expression in CHMp‐13a cells (a–c). Cells were treated with different concentrations (0, 40, 80, and 160 μg/ml) of GPE for 28 h. Relative abundance of mRNA was obtained by normalization to *GAPDH* expression. Each data point indicates the mean of six separate experiments ± SD. Statistical differences were analyzed using Kruskal Wallis followed by Dunn's multiple comparisons post hoc tests. Significant differences from the control are indicated: ^∗^ *p* < 0.05; ^∗∗^ *p* < 0.01

**FIGURE 7:**
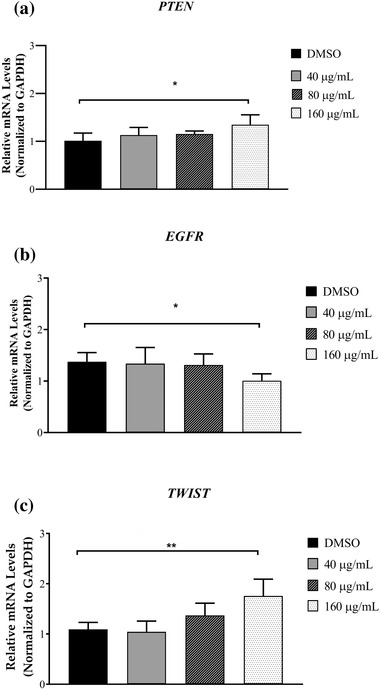
Effects of *Gynura procumbens* leaves extract (GPE) on *PTEN*, *EGFR*, and *TWIST* gene expression in CHMp‐5b cells (a–c). Cells were treated with different concentrations (0, 40, 80, and 160 μg/ml) of GPE for 28 h. Relative abundance of mRNA was obtained by normalization to *GAPDH* expression. Each data point indicates the mean of six separate experiments ± SD. Statistical differences were analyzed using Kruskal Wallis followed by Dunn's multiple comparisons post hoc tests. Significant differences from the control are indicated: *^∗^p* < 0.05; ^∗∗^ *p* < 0.01

The expression level of *PTEN* mRNA in CHMp‐5b cells that were treated with 160 μg/ml of GPE for 28 h was significantly greater compared to the control (Figure 7a). However, the *PTEN* mRNA expression level in CHMp‐13a cells was not increased in any treatment group (Figure 6a). The results indicate that GPE could significantly increase *PTEN* expression in CHMp‐5b cells in a dose‐dependent manner.

The mRNA levels of *EGFR* in CHMp‐13a cells that were treated with 40, 80, and 160 μg/ml of GPE were significantly lower compared to the control (Figure 6b). In CHMp‐5b cells, the mRNA level of *EGFR* was significantly lower than control when they were treated with 160 μg/ml of GPE (Figure 7b). Thus, GPE could be a promising inhibitor of *EGFR* mRNA expression in both CHMp‐13a and CHMp‐5b cells.

The relative mRNA expression levels of the *TWIST* gene between treated and untreated CHMp‐13a cells were not significantly different (Figure 6c). Surprisingly, the expression mRNA level of this gene in the CHMp‐5b cells that were treated with 160 μg/ml of GPE was significantly increased compared to control (Figure 7c).

### 3.5 Protein expression by western blot analysis

**FIGURE 8:**
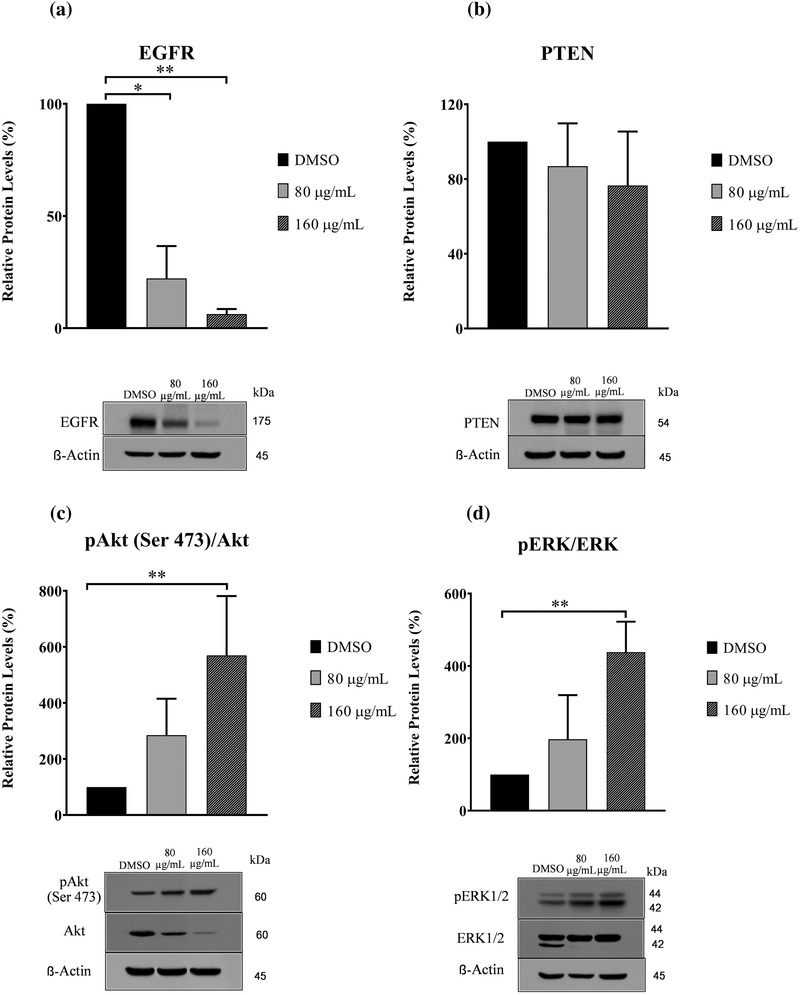
Effects of *Gynura procumbens* leaves extract (GPE) on protein expression of epidermal growth factor receptor (EGFR), phosphatase and tensin homolog (PTEN), AKT, extracellular signal‐regulated kinase 1/2 (ERK1/2), phosphorylated AKT, and phosphorylated ERK1/2 in CHMp‐13a cells (a–d). Cells were treated with different concentrations (0, 80, and 160 μg/ml) of GPE for 28 h. Relative abundance of EGFR and PTEN protein were normalized to ß‐actin. The phosphorylation levels of AKT and ERK1/2 were normalized to the total AKT and ERK1/2, respectively. Each data point indicates the mean of three separate experiments ± SD. Statistical differences were analyzed using a Kruskal Wallis followed by Dunn's multiple comparisons post hoc tests. Significant differences from the control are indicated: *^∗^p* < 0.05; ^∗∗^ *p* < 0.01

**FIGURE 9:**
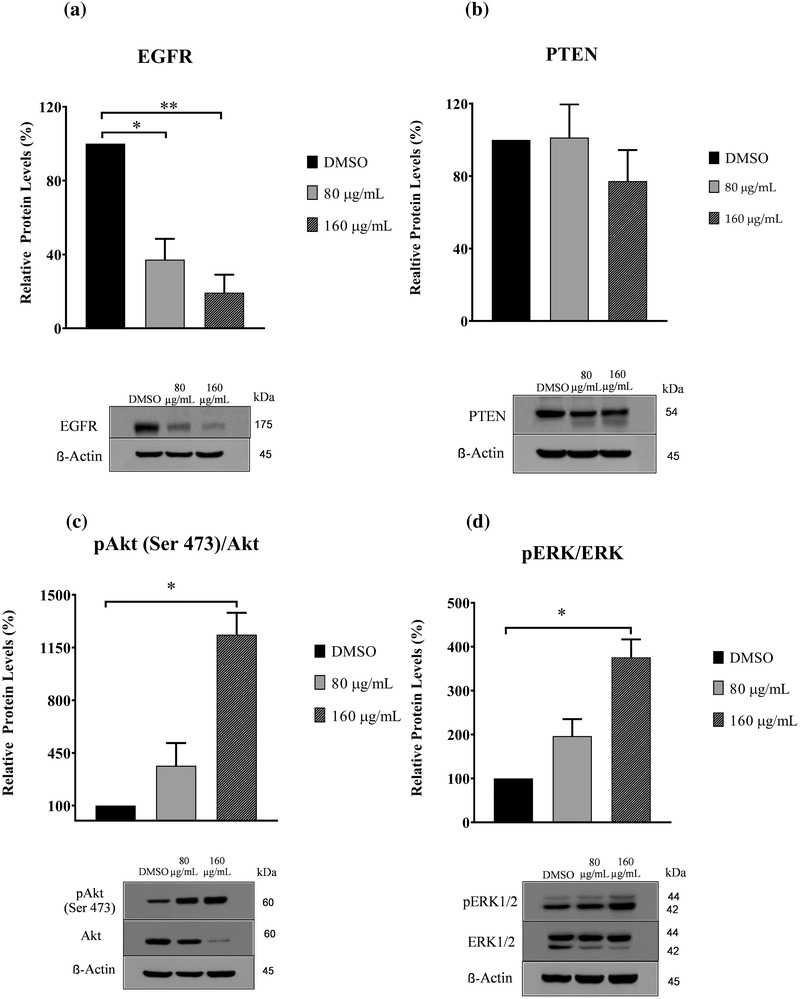
Effects of *Gynura procumbens* leaves extract (GPE) on protein expression of epidermal growth factor receptor (EGFR), phosphatase and tensin homolog (PTEN), AKT, extracellular signal‐regulated kinase 1/2 (ERK1/2) phosphorylated AKT, and phosphorylated ERK1/2 in CHMp‐5b cells (a–d). Cells were treated with different concentrations (0, 80, and 160 μg/ml) of GPE for 28 h. Relative abundance of EGFR and PTEN protein were normalized to ß‐actin. The phosphorylation levels of AKT and ERK1/2 were normalized to the total AKT and ERK1/2, respectively. Each data point indicates the mean of three separate experiments ± SD. Statistical differences were analyzed using Kruskal Wallis followed by Dunn's multiple comparisons post hoc tests. Significant differences from the control are indicated: ^∗^ *p* < 0.05; ^∗∗^ *p* < 0.01

To elucidate the possible mechanisms of GPE, the protein expression levels of EGFR and PTEN were investigated by western blot analysis in both CMC cell lines. Western blot analysis revealed that levels of EGFR protein dramatically decreased in the CHMp‐13a and CHMp‐5b cell lines after all the GPE treatments (Figures 8a and 9a), whereas PTEN protein did not significantly change in treated CHMp‐13a or CHMp‐5b cells (Figures 8b and 9b). Moreover, we also examined the effects of GPE on AKT and ERK1/2 which are downstream signalling proteins in pathways of EGFR pathway, AKT and ERK1/2 signalling. The active phosphorylation levels of AKT (Ser473) and ERK1/2 (Thr202/Tyr204) and the total expression of proteins in the two cell lines treated were measured by western blotting. We found that GPE significantly reduced total AKT protein levels but increased the phosphorylation of AKT at Ser473 (Figures 8c and 9c) and ERK1/2 at Thr202/Tyr204 (Figures 8d and 9d) in both CMC cell lines.

## 4 DISCUSSION

In female dogs, mammary cancer is the most frequently diagnosed tumour. Approximately 80% of CMC cases have recurrence that result in death (Baba & Catoi, 2007). There are a few studies on the efficacy of adjuvant therapy in reducing recurrence and prolonging survival for CMC cases (Karayannapoulou & Lafioniatis, 2016). The efficacy of treatments in this cancer varies greatly depending on the stage of cancer progression, genetic predispositions, and chemotherapeutic tolerance (Jemal et al., 2008). The development of new therapeutic agents is needed for the improvement of animal patient outcomes. A large number of herbal medicines have been discovered (Moraes et al., 2017). Natural plant crude extracts have been widely used in traditional Thai medicine for many diseases, especially cancers (Kummalue et al., 2014; Lueangamornnara et al., 2017; Poofery et al., 2020). However, the knowledge on herbal medicine in CMCs is limited. The efficacy of medical plants in CMC therapy needs to be investigated in much more detail (Gultiken et al., 2015; Sefidabi et al., 2017).

GP, commonly known as longevity spinach, is traditionally used as a treatment in many countries, especially in Southeast Asia (Tan et al., 2016). The in vitro anti‐cancer effects of GP extract have previously been reported in various human cancers, such as colon cancer, osteosarcoma, glioblastoma, and breast cancer (Hew et al., 2013; Rohin et al., 2018; Teoh et al., 2016; H. Wang et al., 2013). The anti‐cancer activities of GP have been examined on several carcinogenic phenotypes, including cell proliferation, metastasis, and angiogenesis. However, the anti‐cancer effect of GP against CMCs has not been investigated.

In this study, the cytotoxicity effect of GPE was investigated on CHMp‐13a and CHMp‐5b cell lines. Our results indicated that cells exposed to GPE lose their ability to proliferate in a time‐ and dose‐dependent manner. The result suggests that GPE has the potential to inhibit the growth of CMC cells in vitro. The wound healing and transwell migration assays were performed to investigate the effect of GPE on CHMp‐13a and CHMp‐5b cell migration. The results of wound healing and transwell migration assays revealed that GPE could significantly suppress both CMC cells migration in a dose‐dependent manner in vitro.

Apoptosis is a mode of programmed cell death that is mediated by caspase family members (Lüthi & Martin, 2007). They are categorized as initiator (caspase 2, 8, 9, and 10) and effector caspases (caspase 3, 6, and 7) (Riedl & Shi, 2004). The initiator caspases are responsible for initiating caspase activation cascades, and the effector caspases are thought to be responsible for the cell destruction during apoptosis. In particular, the activation of the effector caspase 3/7 is involved in the late stage of cell death (Elmore, 2007). The increase in caspase 3/7 activity observed in both CMC cells treated with GPE suggests that GPE induced caspase 3/7‐dependent apoptosis within 28 h after treatment.

From the relative mRNA and protein expression analysis, it was observed that the mRNA and protein expression level of EGFR was significantly lower in both CHMp‐13a and CHMp‐5b cells treated with GPE compared to the untreated cells. EGFR in malignant tumours is thought to be a promoter of cancer cell proliferation, invasion, and metastasis (Masuda et al., 2012). High expression of EGFR in CMC can increase angiogenesis and metastasis (Guimaraes et al., 2014). Molecular inhibition of EGFR expression in cancer cells could inhibit the epidermal growth factor that induces increased proliferation, migration, and apoptosis (Khan et al., 2006; Manupati et al., 2017). To investigate the mechanism of action of GPE on EGFR‐mediated signal transduction, we examined the effect of GPE on AKT/ ERK signalling pathway. We found that GPE did not inhibit the active phosphorylation of AKT and ERK1/2 levels. These results suggest that GPE inhibits the cell proliferation and migration and induces apoptosis of CHMp‐13a and CHMp‐5b cell lines via the inhibition of EGFR, but not related to the downstream AKT/ERK1/2 signalling pathway. The inhibition of EGFR expression by GPE on CMC cells may down‐regulate to other downstream signalling pathways. Moreover, the up‐regulation of both AKT and ERK1/2 phosphorylation level is possibly related to cross‐talk between signalling pathways. Additionally, it must be noted that we observed that GPE caused a reduction of ERK2 in both cell lines with more pronounced effect in CHMp‐13a, suggesting that GPE may induce ERK2 degradation in these cell lines. Because mitogen‐activated protein kinase kinase kinase 1 (MEKK1), which is an upstream activator of ERK, has been shown to induce ERK degradation through ubiquitin‐dependent pathway (Lu et al., 2002) we reason that ERK2 degradation by GPE may be due to MEKK1‐mediated ubiquitination‐dependent degradation. Therefore, future studies are required to investigate the mechanism of action of GPE on CMC cells via inhibition of EGFR.

PTEN is an important tumour‐suppressor that modulates multiple biological processes, including apoptosis, cell proliferation, and cell growth (Tong et al., 2016). In CMC, the reduced PTEN expression significantly correlated with lymph node metastases, distant organ metastasis, tumour differentiation, tumour recurrence, and lower overall survival rate (Ressel et al., 2009). Moreover, loss of PTEN expression is also frequently observed in chemotherapeutic resistant breast cancers (Tanic et al., 2012). On the other hand, the over‐expression of PTEN promotes cell apoptosis and downstream effects involving the PI3K/AKT intracellular signalling pathway that promotes cell proliferation, cell survival, and angiogenesis (Nordin et al., 2017). In this study, significantly greater *PTEN* mRNA expression levels in CHMp‐5b cancer cells treated with GPE (at a concentration of 160 μg/ml) compared to control was observed. However, we did not observe the significantly greater *PTEN* mRNA expression level in CHMp‐13a cells compared to control. In contrast, western blot analysis revealed that the PTEN protein level did not significantly change in both CMC cell lines. However, the PTEN protein in both CMC cells was separated into two bands by western blot upon treatment with GPE. The additional band below PTEN band observed in our experiment may be a result from PTEN degradation. Phosphorylation has been shown to promote stability but inhibits activity of PTEN (Xu et al., 2014). It is to be noted that although GPE did not alter the levels of PTEN in both cells line, it may increase PTEN degradation as evidenced by a lower molecular weight PTEN band in cells treated with GPE. We reason that GPE may reduce PTEN phosphorylation thus increasing its degradation. However, the levels of full length PTEN were not affected by the treatment, so we believe that the overall PTEN activity may not alter in our experimental condition. This result suggests that PTEN may not play a molecular role in the anti‐cancer effect of GPE against both CMC cells via PI3K/AKT signalling pathway. It is likely that GPE may induce apoptosis, proliferation, and growth in CHMp‐13a and CHMp‐5b cancer cells using a different signalling pathway

*TWIST*, one of the epithelial mesenchymal transition (EMT) associated genes, is involved in tumour invasion and metastasis. The over‐expression of *TWIST* remarkably enhances the invasive and metastatic capacities of CMC cells by inducing EMT. However, many studies reported that there are multiple drivers involved in the EMT process, such as the down‐regulation of E‐cadherin expression and the up‐regulation of several EMT associated genes (*TWIST*, *SNAIL*, *SLUG*, and *ZEB*) (Raposo‐Ferreira et al., 2018; D. Wang et al., 2018). In this study, no difference in *TWIST* mRNA expression level between treated and untreated CHMp‐13a cancer cells with GPE were observed. Conversely, the mRNA expression level of *TWIST* in GPE treated CHMp‐5b cancer cells was significantly greater compared to control. Our results propose that GPE may not inhibit the migration and invasion of CMC cells via *TWIST*‐mediated EMT.

The cell growth inhibition and apoptotic effect of GPE may be related to its major components of flavonoids such as kaempferol, astragalin, and quercetin (Rosidah et al., 2008). Flavonoids are most abundantly occurring polyphenols in plants that are the most promising anti‐cancer agents (Batra & Sharma, 2013). Oncology researchers have reported that flavonoids can be used as a single agent or in combination with other chemotherapy procedures to control the growth of many types of human cancer cells (Agustina et al., 2006; Chahar et al., 2011; Kopustinskiene et al., 2020; Purba & Astuti, 2013). Furthermore, similar studies have reported the anti‐proliferation, anti‐migration, and apoptosis inducing effects of kaempferol, astragalin, and quercetin on human breast cancer cells (Chiang et al., 2013; Li & Xue, 2019; Panche et al., 2016; Salehi et al., 2020; X. Wang et al., 2019; Yaghoubi & Moradi, 2020). Their anti‐cancer effects are associated with disrupting the cell cycle via the induction of arrest at G2/M phase (Eun & Woong, 2008), down‐regulation of cell migration proteins expression, such as EGFR, matrix metalloproteinase 2 (MMP‐2), MMP‐9, and vascular endothelial growth factor, inducing autophagy by inactivating the Akt‐mTOR pathway (Balakrishnan et al., 2016; Jia et al., 2018), and inducing apoptosis via p53 phosphorylation in human breast carcinoma cells (Eun & Woong, 2008). It is also of significant importance to note that there are similarities between human and dog mammary cancer which include hormonal dependence, the pattern of neoangiogenesis, spontaneous development, and metastatic behaviour. Thus, the anti‐carcinogenic effect on CMC may also relate to the crude extract of GP which consists of polyphenol compounds such as kaempferol, astragalin, and quercetin.

Several studies have reported the in vitro and in vivo anti‐cancer activities of crude extract of GP in human cell lines and animal models. The GPE at IC_50_ 90 μg/ml could increase the effectiveness of doxorubicin on T47D human breast cancer cell (Jenie et al., 2007), whereas the concentration of 200 μg/ml showed the synergism effect when combined with 5‐fluorouracil on WiDr human colon cancer cell (Nurulita et al., 2011). Furthermore, treatment of GPE at concentration of 300 mg/kg by oral administration significantly inhibited the carcinogenesis of dimethylbenz(a)antracene (DMBA) on male rat liver and on female rat mammary glands induced by DMBA without any adverse effects (Hamid, 2009; Nisa et al., 2012). In addition, the acute toxicity studies showed that the high dose of GPE at concentration 5 g/kg did not cause any toxicity in Sprague‐Dawley rats after 14 days of oral administration (Zahra et al., 2011). These efficacy and safety data of GPE suggest that this herbal extract has a potential to be developed as a single agent or in combination with other chemotherapy in the treatment of CMC. However, further studies need to explore the mechanism and interaction of combined treatment for CMC. Furthermore, the additional studies using purified bioactive compounds of GPE are required to provide a better understanding of the effect of GPE on signalling pathways altered by oncogene expression in CMC.

## 5 CONCLUSION

**FIGURE 10:**
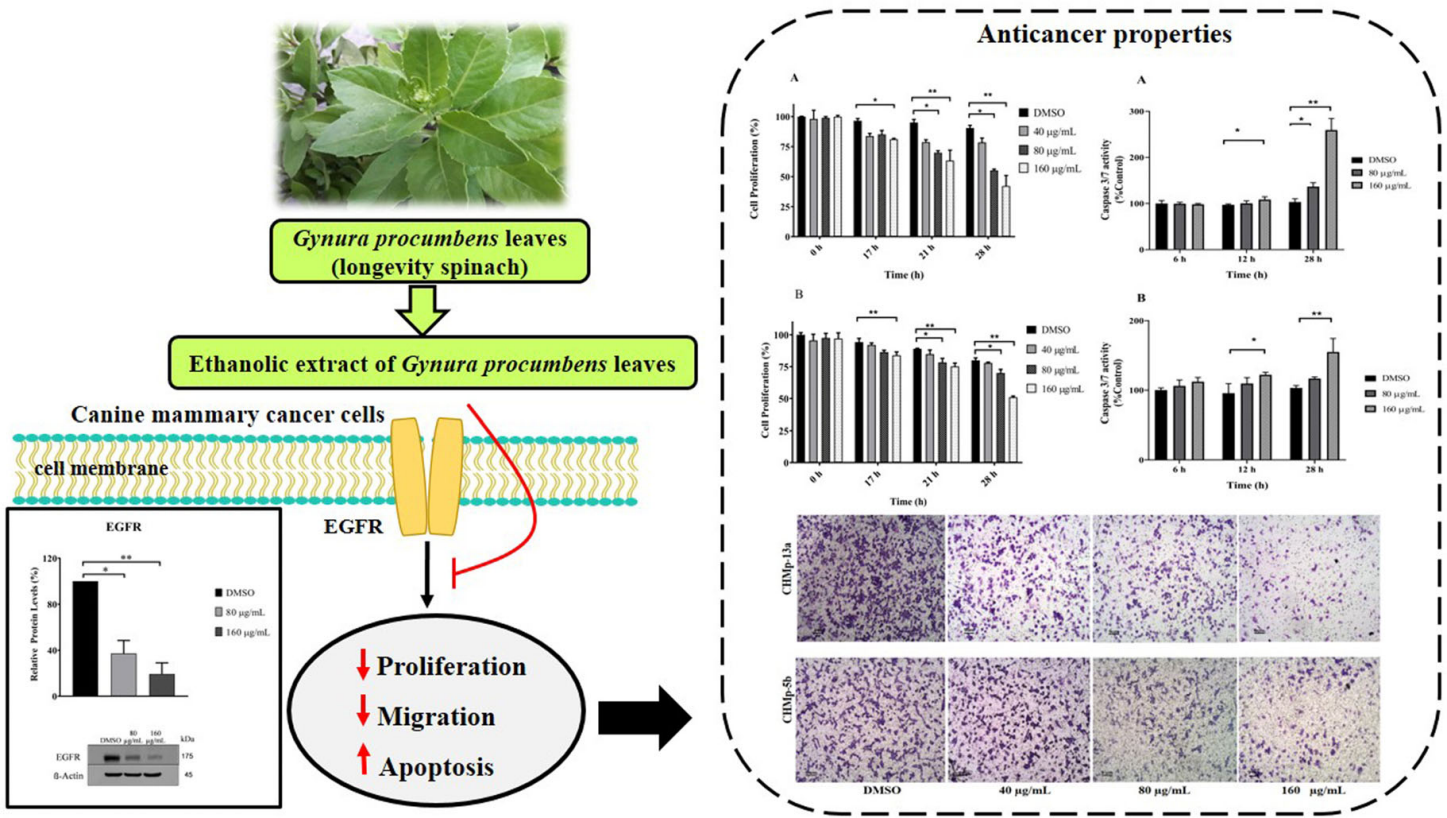
In vitro anti‐cancer properties of *Gynura procumbens* leaves extract (GPE) against canine mammary cancer (CMC) cell lines by inhibiting epidermal growth factor receptor (EGFR) signalling pathway

To the best of our knowledge, this is the first study to evaluate the anti‐cancer effect of ethanolic extract of GP in CMC cell lines. The results have shown that this herbal extract significantly reduces cancer cell proliferation and migration, induces cancer cell apoptosis, and decreases the expression of EGFR in vitro (Figure 10). These findings indicate that GPE has a high potential in terms of cancer therapy in dogs through the inhibition of the EGFR signalling pathway. However, additional studies are needed to clarify the molecular mechanisms of GPE via EGFR signalling pathway underlying anti‐cancer activity in CMCs. Moreover, further studies elucidating the mechanisms of action of the bioactive compounds are needed for the development of this herbal product and are also warranted to determine how GPE can be used as an effective complementary therapy combined with chemotherapy for CMC.

## CONFLICT OF INTEREST

The authors declare no conflict of interest.

## ETHICS STATEMENT

No ethical approval was required, as this study required no animal experiments.

## AUTHOR CONTRIBUTIONS

*Project administration, methodology, investigation, data curation, validation, writing‐original draft, and writing‐review & editing*: Usuma Jermnak. *Methodology, investigation, data curation, validation, and writing‐review & editing*: Wachiraphan Supsavhad. *Conceptualization*: Sunee Kunakornsawat. *Conceptualization and resources*: Tassanee Jaroensong. *Methodology, investigation, data curation, and writing‐review & editing*: Piyajit Watcharasit. *Methodology and investigation*: Daranee Visitnonthachai. *Resources*: Selapoom Pairor. *Methodology and Resources*: Napasorn Phaochoosak.

## Data Availability

The data that support the findings of this study are available from the corresponding author upon reasonable request.
